# Clinical Significance of Mesiobuccal and Distobuccal Canal Variations in Maxillary Molars: A Case Series and a Mini Review

**DOI:** 10.1155/crid/5581317

**Published:** 2025-11-12

**Authors:** Mohsen Aminsobhani, Somayeh Majidi

**Affiliations:** ^1^Endodontics, Dental School, AJA University of Medical Sciences, Tehran, Iran; ^2^Tehran University of Medical Sciences, Tehran, Iran; ^3^Department of Endodontics, School of Dentistry, Tehran University of Medical Sciences, Tehran, Iran

**Keywords:** anatomical variations, canal morphology, cone beam computed tomography (CBCT), DB2 canal, endodontics, maxillary molars, MB3 canal, root canal treatment

## Abstract

Endodontic treatment success is contingent upon the comprehensive identification and management of all root canals within a tooth's complex anatomy. This is particularly challenging in maxillary molars, which exhibit significant variability in canal morphology. While the presence of two canals in the mesiobuccal (MB) root is well documented, the occurrence of a third canal (MB3) is less common yet clinically relevant. Additionally, the distobuccal (DB) root, typically containing a single canal, may occasionally harbor a second canal (DB2). This case series presents clinical cases that highlight the prevalence and significance of MB3 and DB2 canals in maxillary molars. The findings underscore the necessity for meticulous canal detection using advanced imaging techniques such as cone beam computed tomography (CBCT), as traditional diagnostic methods may overlook these anatomical variations. By sharing these cases, we aim to enhance awareness and encourage further research into the implications of these additional canals on endodontic treatment outcomes.

## 1. Introduction

Endodontic treatment success depends on the thorough identification and management of all root canals within a tooth's complex anatomy [1]. Overlooking any canal can lead to persistent infection, continued clinical symptoms, and the need for retreatment or extraction [2]. This challenge is particularly pronounced in maxillary molars, which are known to have variable canal morphology [3]. So, the identification of these additional canals is crucial for successful endodontic treatment of these teeth [2].

Although the presence of two canals in the mesiobuccal (MB) root is well documented, the occurrence of a third mesiobuccal canal (MB3) is less common but clinically significant. Similarly, a distobuccal (DB) root, which typically contains a single canal, may occasionally harbor a second distobuccal canal (DB2) [4].

The occurrence of a third canal in the MB root has been reported to range from 1.3% to 2.4% in various studies depending on population and the detection method employed, indicating that while it is infrequent, it is not negligible [4]. Some reports suggest that the MB3 canal can be present in approximately 15% of cases under certain conditions, emphasizing the need for thorough examination during treatment [4, 5] Although specific prevalence rates for DB2 are less frequently discussed in literature compared to MB3, corresponding figures for the distobuccal root (DB2) were 5.10% (ex vivo results) and 3.75% (clinical results), resulting in a high rate of missed diagnosis [6].

Traditional diagnostic methods, while essential, may not always be sufficient to detect these elusive canals [7]. Recent advancements in imaging technology, particularly cone beam computed tomography (CBCT), have significantly improved the detection of complex canal systems [8].

CBCT provides three-dimensional visualization, offering enhanced detection sensitivity, which explains why many recent publications report increased identification of MB3 and DB2 canals [5, 9–12]. Similarly, the use of dental operating microscopes (DOMs) has facilitated intraoperative detection of smaller or calcified canals that are otherwise difficult to visualize under unaided vision [1, 10, 11, 13–16]. Using CBCT in retreatment cases, especially when there is unexplained persistent periapical disease, is recommended to rule out missed canals.

This case series is aimed at contributing to the existing literature by presenting four clinical cases that highlight the role of advanced technologies in the detection of additional canals in maxillary molars. Through detailed analysis and discussion, these cases underscore the importance of meticulous canal identification and management strategies in endodontics, ultimately contributing to improved clinical outcomes and long-term treatment success.

## 2. Materials and Methods

### 2.1. Case Selection

Patients presenting with maxillary first or second molars requiring primary or secondary endodontic treatment, with suspected or confirmed anatomical variations (notably additional canals such as MB3 or DB2), identified via clinical examination, CBCT imaging, or intraoperative findings, were included. Exclusion criteria comprised teeth with prior surgical interventions (e.g., apicoectomy or root resection), nonrestorable teeth due to extensive structural damage, severe periodontal disease, root fractures, systemic health conditions contraindicating endodontic therapy, or lack of patient consent.

### 2.2. Ethical Approval

Written informed consent was obtained from the patient, authorizing the use of clinical data for academic publication, with full understanding of its scope and implications. Prior to treatment, all patients were informed about the potential risks and benefits and made an informed decision to proceed. The study adhered to the Declaration of Helsinki, and patient anonymity and confidentiality were strictly maintained.

### 2.3. Diagnostic and Treatment Protocol

This case series was prepared in accordance with the Guidelines for Reporting the Quality of Clinical Case Reports in Endodontics (PRICE), which promote comprehensive, transparent, and standardized documentation of clinical findings, diagnostics, treatment procedures, and outcomes [17]. Four Iranian patients were treated under uniform conditions by a single experienced endodontist with over 30 years of clinical expertise. Prior to treatment, risks, benefits, and alternatives were discussed with patients, and written informed consent was secured. Local anesthesia was administered by buccal infiltration using 1.8 mL of 2% lidocaine with 1:80,000 epinephrine (Darupakhsh, Tehran, Iran). After rubber dam isolation, access cavities were prepared under magnification from a DOM (Magna Labomed, Labo America Inc., United States). Canal scouting employed rotary instruments selected according to individual canal anatomy. While rotary systems varied per case, the irrigation protocol was consistent: copious 5.25% sodium hypochlorite (NaOCl) activated ultrasonically (NSK Varios, Nakanishi Inc., Tochigi, Japan) to maximize disinfection. Working length was determined by an electronic apex locator (Dempex, DEM Ltd., Barnstaple, United Kingdom) and confirmed radiographically. Cleaning and shaping were performed under DOM magnification using the crown-down technique to ensure meticulous instrumentation. In all retreated cases, eucalyptol (Cerkamed, Poland) was used as the solvent for the removal of root canal filling materials during retreatment.

Obturation techniques were individualized based on canal morphology and clinical considerations, using contemporary materials and methods to achieve a hermetic seal. Teeth were temporarily restored at the end of each session to maintain coronal seal integrity and prevent reinfection. Postoperative clinical and radiographic assessments confirmed successful outcomes, with resolution of symptoms and evidence of periapical healing in all cases.

## 3. Clinical Cases

### 3.1. Case 1 (Endodontic Management of Symptomatic Irreversible Pulpitis in a Maxillary Left Second Molar With MB3 Canal)

#### 3.1.1. Case History/Examination

**Figure 1:**
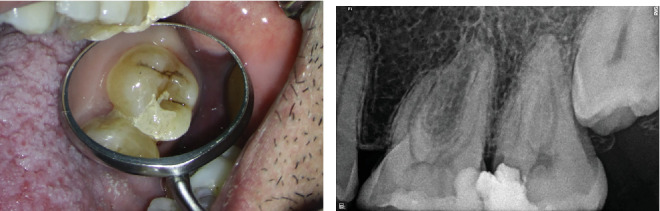
(a) Intraoral photographs of Tooth #15 prior to treatment. (b) Periapical radiograph captured before initiating root canal therapy.

A 30-year-old male patient was referred to the dental clinic for evaluation and treatment of the maxillary left second molar. The patient reported experiencing severe, throbbing pain localized to the upper molars for a duration exceeding 3 weeks. Notably, the patient's medical history was noncontributory, posing no additional risk factors for dental treatment. Upon clinical examination, the affected tooth was found to have a restorative material (Figure 1a). Palpation and percussion tests revealed no discomfort; however, the tooth exhibited significant hypersensitivity to cold stimuli. Radiographic assessment revealed a defective restoration located on the mesial aspect of the tooth, extending toward the pulpal outline (Figure 1b).

#### 3.1.2. Diagnosis/Treatment

Based on the clinical and radiographic findings, a diagnosis of symptomatic irreversible pulpitis was established, necessitating endodontic intervention.

**Figure 2:**
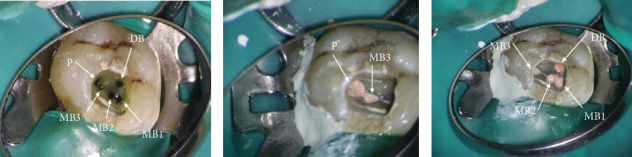
Intraoperative microscopic examination of the pulp chamber of Tooth #15. (a) Identification and localization of canals and three distinct orifices in MB root. (b) Postobturation view of MB3 and P canals. (c) Postobturation view of MB1, MB2, MB3, and DB canals.

After obtaining informed consent, the patient underwent root canal therapy following the administration of local infiltration of anesthetic solution; 1.8 mL of 0.2% lidocaine with 1/80,000 epinephrine (Darupakhsh, Tehran, Iran) for local infiltration was injected. After isolating with rubber dam, an access cavity was prepared with a high-speed diamond round bur number 2 (Jota AG, Rüthi, Switzerland) and continuous water spray with the aid of a DOM at a magnification of 10x (Magna Labomed, Labo America Inc., United States), which facilitated a detailed visualization of the root canal anatomy. The examination revealed two orifices in the MB root, one orifice in the DB root, and one orifice in the palatal (P) root. The provisional working length was determined by an electronic apex locator (Dempex, DEM Ltd., Barnstaple, Devon, England) and confirmed by radiography. Cleaning and shaping of all canals were initiated by Superfile III Denco (Denco, Shenzhen, China) rotary instruments. During the instrumentation of the MB2 canal, notable bleeding was observed, prompting additional irrigation and careful instrumentation. Guided by the microscope, an orifice was identified adjacent to the P canal.  Figure 2a illustrates the three distinct orifices present in the MB root.

Upon successful negotiation of the MB3 canal, the bleeding observed from the MB2 canal subsided. Utilizing a size 10 K-file to navigate the MB canal, it was determined that the two canals within the MB root converged before their exit, forming two distinct foramina. All canals were meticulously prepared using rotary files up to 25 4% except P up to 30 4% employing a crown-down technique. The canals were irrigated with copious amounts of 5.25% NaOCl for thorough disinfection, ultrasonic agitation with U-file ultrasonic tip size 15, 0.02 taper (NSK Varios, Nakanishi Inc., Tochigi, Japan) was employed. The configuration of the MB canal revealed three orifices (3-2-2); meanwhile, both DB and P canals exhibited Type I configurations (Figure 2) consistent with Ahmed et al. [18] canal coding 16 MB3-2-2 DB1 P1.

**Figure 3:**
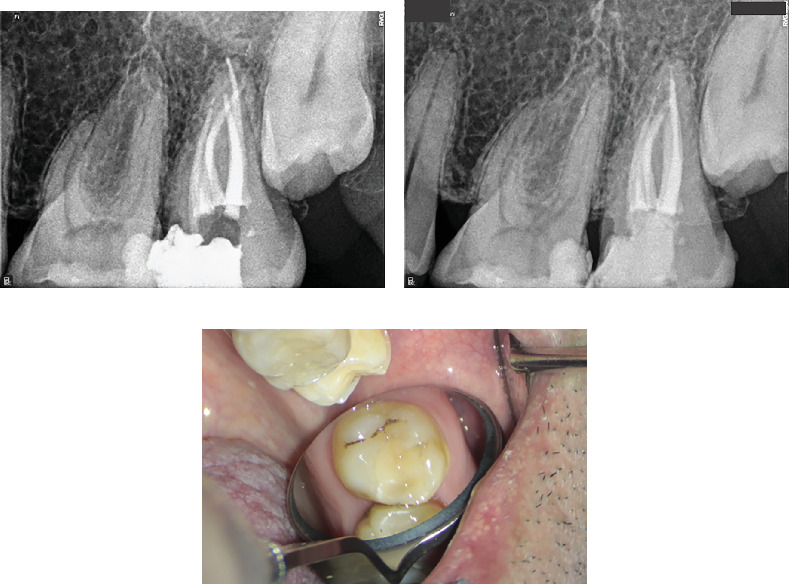
(a) Postoperative radiograph illustrating the obturated mesiobuccal (MB), distobuccal (DB), and palatal (P) canals. (b) Periapical radiograph at 7 years and 4 months of follow-up demonstrating normal periradicular structures with no evidence of pathological changes. (c) Intraoral photograph of the treated tooth at 7 years and 4 months of follow-up, showing healthy surrounding soft tissues and an intact permanent restoration.

Due to intracanal bleeding, calcium hydroxide paste (Merck, Darmstadt, Germany) was placed as an intracanal medicament. After 1 week, complete resolution of pain was achieved and obturation was completed using a sealer-based technique with gutta-percha (GP) (META, Chungbuk, South Korea) and Endoseal MTA sealer (Maruchi, Wonju, Korea). The access cavity was temporarily restored with zinc oxide powder (Zonalin, Kemdent, Wiltshire, United Kingdom).  Figure 3a illustrates the postoperative radiograph.

The treated tooth was subsequently referred for permanent restoration. Seven years and 4 months post–root canal treatment, the tooth remains functional with no clinical or radiographic signs of pathology (Figure 3b,c).

### 3.2. Case 2 (Endodontic Management of a Missed Canal in a Maxillary Right First Molar)

#### 3.2.1. Case History/Examination

**Figure 4:**
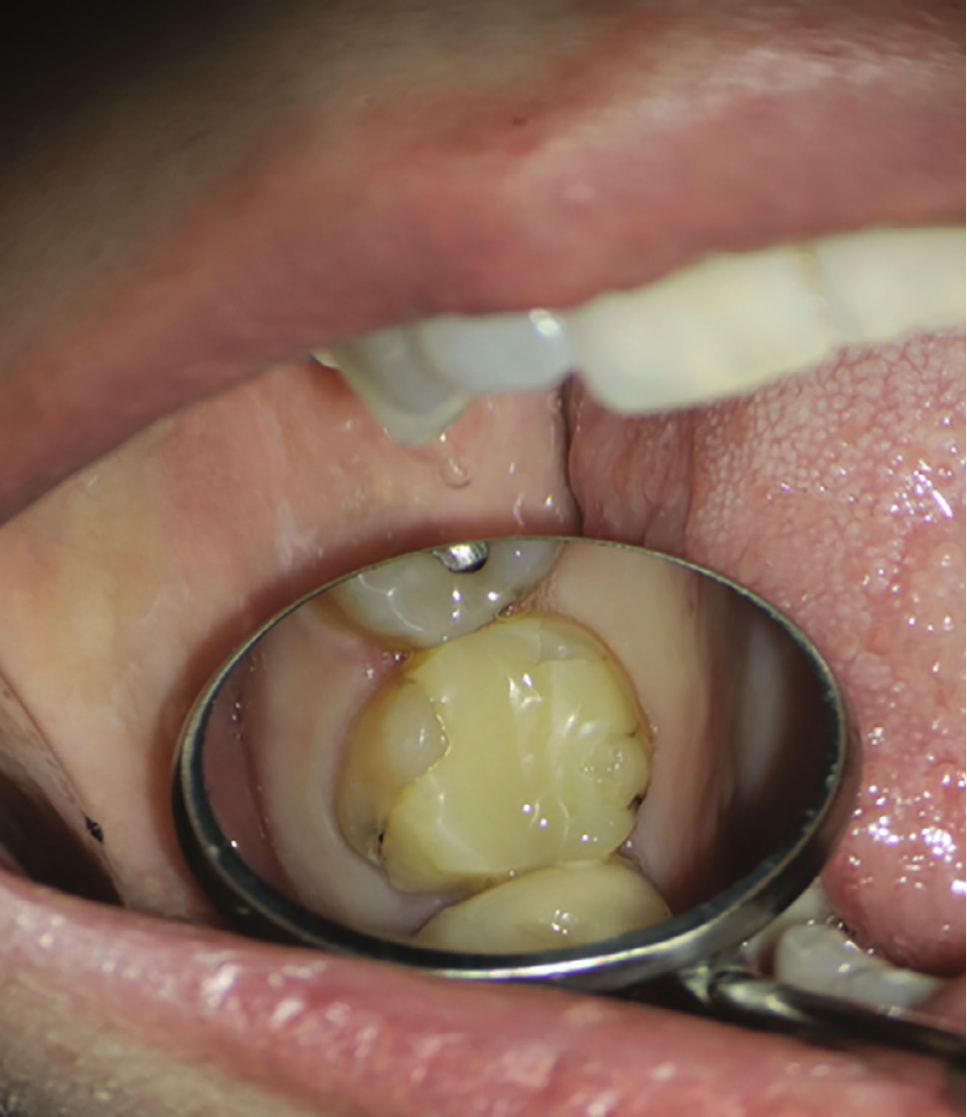
Intraoral photography.

A 36-year-old female patient, with no significant medical history, was referred for dental consultation regarding her previously treated maxillary right first molar (Tooth #3) which was done 2 years ago. Also, Tooth #4 needs treatment. The patient had an abscess and swelling that subsided by using antibiotics (co-amoxiclav) and now in clinical examination had no swelling and has no pain (Figure 4).

**Figure 5:**
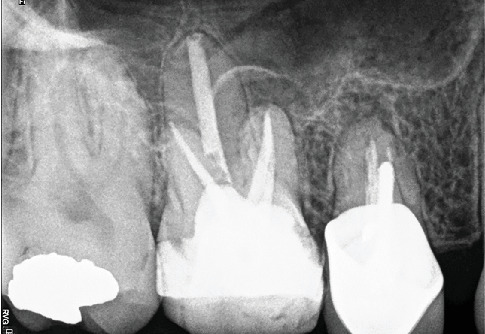
Pretreatment radiographic examination of Tooth #3 revealed the presence of an extensive periapical lesion and also dense obturating material in MB1, MB2, DB, and P canals.

No pain was present, while probing depths around the tooth remained within normal limits, suggesting that periodontal health was intact. Radiographic examination revealed a large periapical radiolucency at the apex of the MB root. The previous root canal treatment appeared well executed, with both MB1 and MB2 canals adequately obturated and exhibiting dense GP filling (Figure 5).

**Figure 6:**
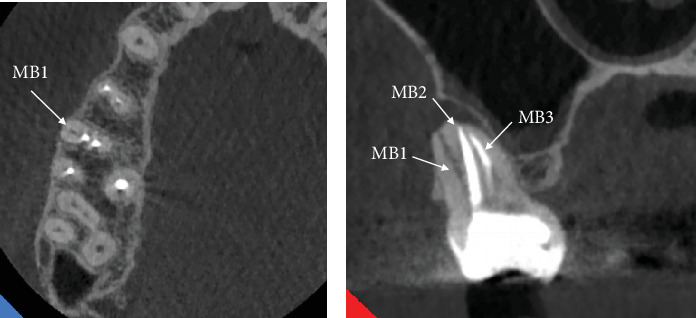
(a) Axial and (b) coronal views highlighting the missed MB1 canal, as indicated by the arrows.

However, the presence of a significant periapical lesion raised concerns regarding potential missed canals or incomplete treatment. To gain further insight into the canal anatomy and periapical condition, a CBCT scan was ordered. The axial view of the CBCT revealed the presence of a missed canal in the MB root, specifically identifying it as the MB1 canal (Figure 6a). Coronal views indicated that both MB1 and MB2 canals shared a common orifice before bifurcating approximately midway down the canal, demonstrating an Ahmed et al. [18] anatomical configuration coding 3 MB2-3-2 DB1 P1 (Figure 6b).

Additionally, a large periapical lesion associated with the MB canal was evident in the coronal view of the CBCT (Figure 6b), indicating ongoing inflammatory processes necessitating intervention.

#### 3.2.2. Diagnosis/Treatment

**Figure 7:**
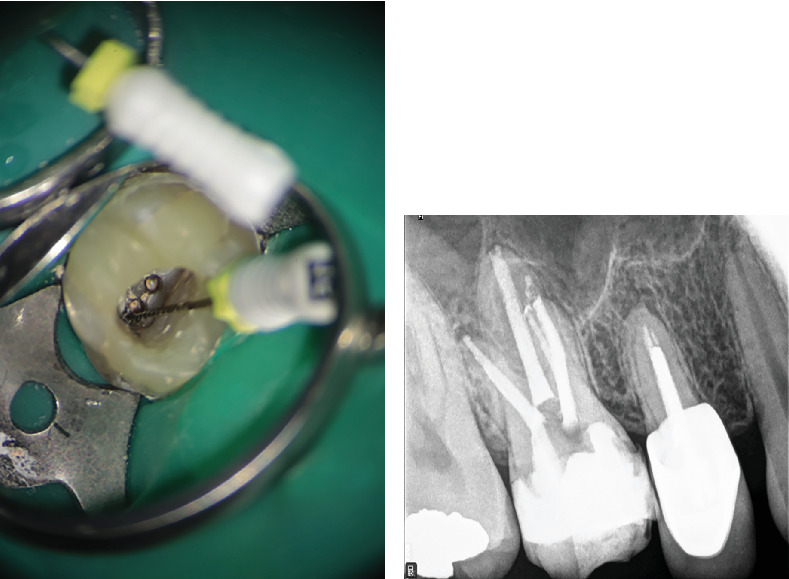
(a) Localization of the newly identified MB1 canal. (b) Obturation of the three distinct MB canals, Ahmed et al. [18] configuration coding 3 MB2-3-2 DB1 P1.

Following the diagnosis of a missed canal and associated periapical pathology, the decision was made to retreat the affected tooth. After obtaining informed consent, local anesthesia was achieved by local infiltration with 2% lidocaine and epinephrine 1:100,000 (Darupakhsh, Tehran, Iran). After isolation with a rubber dam, the restoration was removed with a high-speed diamond round bur number 2 (Jota AG, Rüthi, Switzerland) and continuous water spray under a DOM (Magna Labomed, Labo America Inc., United States). After the removal of the coronal filling material, a drop of eucalyptol (Cerkamed, Poland) was introduced into the canal. A size 15 hand file was then used to create a pathway through the obturation material [19], which was subsequently and meticulously removed from all canals using the Superfile III rotary system (Denco, Shenzhen, China). The newly identified MB1 canal (Figure 7), MB2, and MB3 canal were fully prepared with rotary files (Superfile III Denco, Shenzhen, China) up to 25 4% to ensure adequate cleaning and shaping.

**Figure 8:**
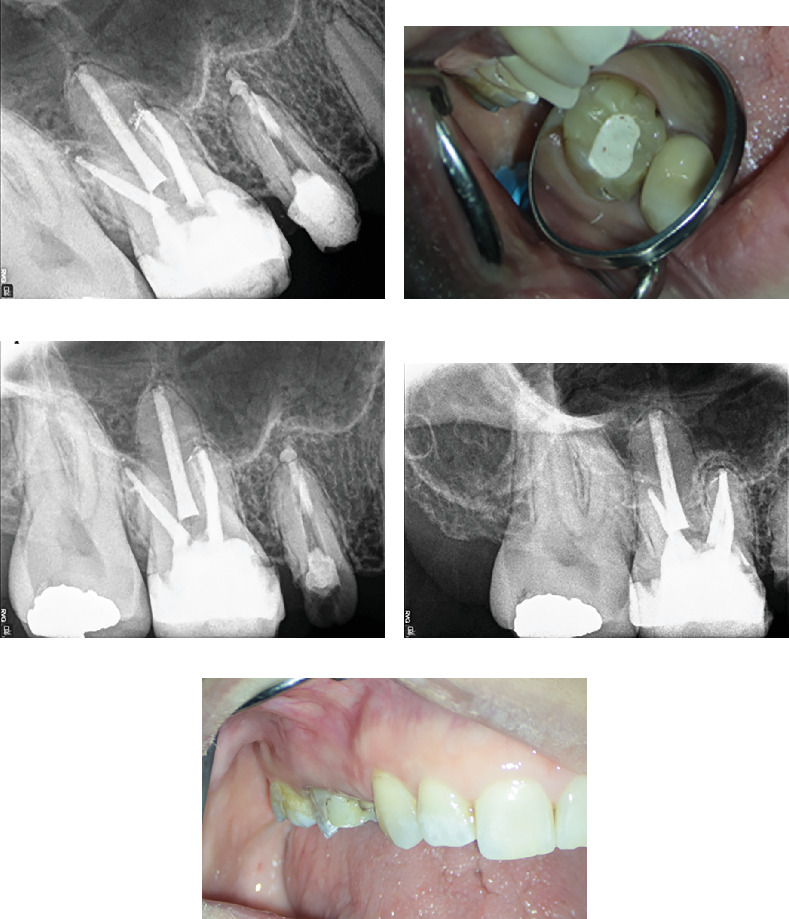
(a) Six-month radiographic follow-up shows complete resolution of the periapical radiolucency, while (b) provides intraoral photography of the treated tooth after 6 months. (c, d) Periapical radiographs at the 2-year and 3-month follow-up reveal normal periradicular structures with no signs of pathology. (e) Intraoral photograph at 2 years and 3 months posttreatment showing stable clinical conditions and absence of pathological changes.

The canals were irrigated copiously with 5.25% NaOCl to facilitate disinfection and removal of debris. After thorough irrigation and agitation with U-file ultrasonic tip size 15, 0.02 taper (NSK Varios, Nakanishi Inc., Tochigi, Japan), the canals were dressed with Ca(OH)2 paste (Merck, Darmstadt, Germany) and chlorhexidine (CHX) 2% (NikDarman, Iran) as an intracanal medicament and temporally restored with intermediate restorative material (IRM) (Dentsply Sirona). At the next appointment, the patient reported being asymptomatic, with no signs of discomfort or swelling. Upon removal of the temporary restoration, the intracanal medicament was irrigated out using NaOCl 2.25%, saline, and EDTA 17% (Master-Dent, Dentonics Inc., United States), also agitated by ultrasonic device (U-file tip size 15, 0.02 taper NSK Varios, Nakanishi Inc., Tochigi, Japan). All canals were dried using sterile paper points (META, Chungbuk, South Korea). The final obturation was completed using lateral compaction techniques with GP (META, Chungbuk, South Korea) and sealer AH-26 (Dentsply, Tulsa Dental, Tulsa, Oklahoma, United States), ensuring complete filling of all canals. The final periapical radiograph confirmed successful obturation of all MB canals, clearly illustrating the separation of MB1 and MB2 in the midcanal region (Figure 7b). The tooth was subsequently restored permanently following successful endodontic treatment. At the 6-month follow-up, the patient remained asymptomatic, and radiographic assessment revealed substantial resolution of the periapical radiolucency, indicative of successful healing and reestablishment of periapical health. The 2-year and 3-month radiographic follow-up demonstrated long-term stability of the treated tooth, with complete resolution of the previous lesion and restoration of normal periapical tissue architecture (Figure 8).

### 3.3. Case 3 (Endodontic Management of a Maxillary Right First Molar With Complex Canal Anatomy)

#### 3.3.1. Case History/Examination

**Figure 9:**
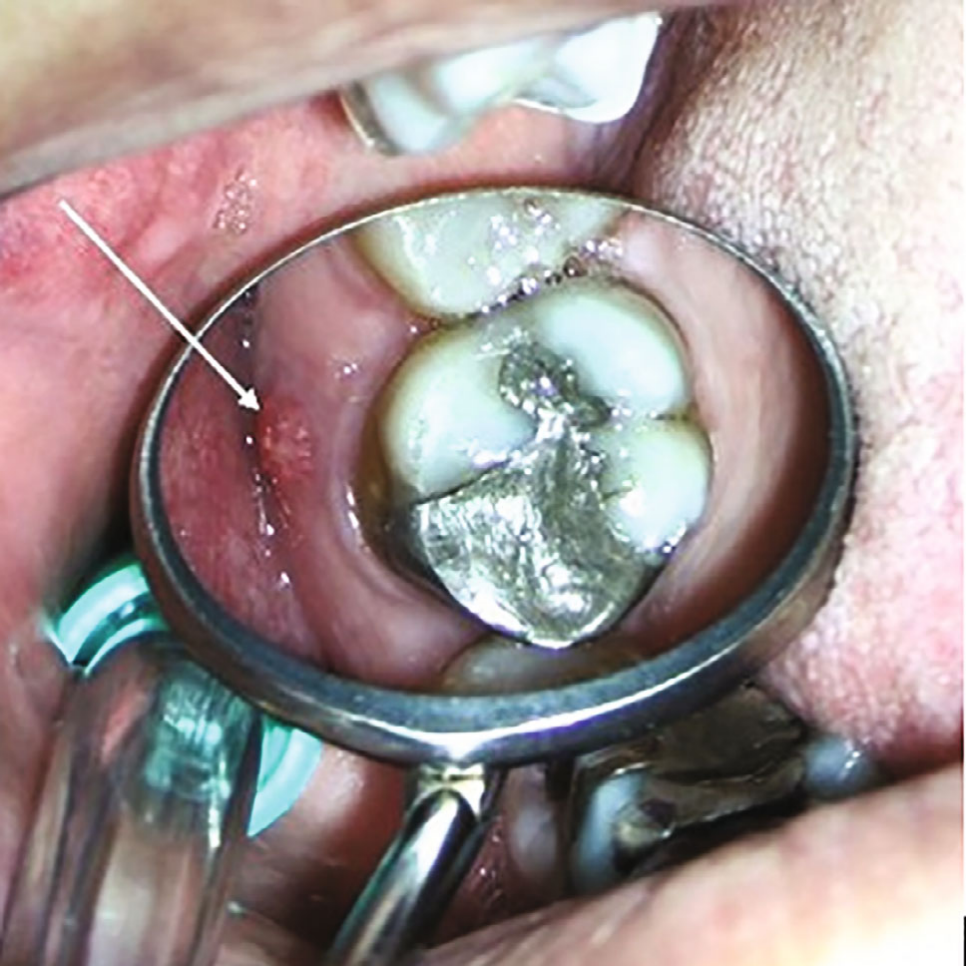
Intraoral photograph of swelling with sinus tract in the buccal vestibule, indicative of an abscess formation as indicated by an arrow.

This case report details the endodontic management of a 43-year-old female patient presenting with complications associated with her maxillary right first molar (Tooth #3). The patient had a medical history of hypothyroidism and was under treatment with levothyroxine Na 50 mg daily. The clinical presentation and radiographic findings indicated the need for further intervention due to previous inadequate root canal treatment. The patient was referred to our dental clinic for evaluation of Tooth #3, which had undergone prior root canal therapy. Clinical examination revealed a large amalgam restoration on the tooth, accompanied by tenderness to percussion and palpation. Additionally, there was notable swelling with a sinus tract in the buccal vestibule, indicative of an abscess formation (Figure 9).

**Figure 10:**
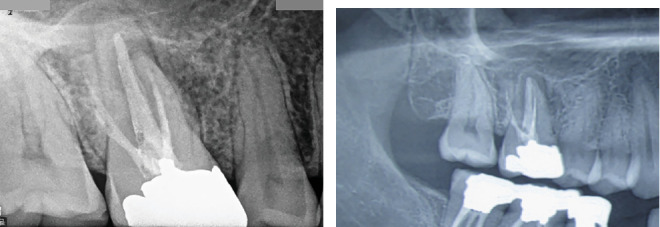
Radiographic evaluation revealed a substantial periapical radiolucency encircling the apices of the MB, DB, and P roots. (a) Periapical view and (b) a part of the panoramic view.

Radiographic assessment demonstrated a significant periapical radiolucency surrounding the apices of the MB, DB, and P roots, suggesting ongoing periapical pathology (Figure 10a,b).

#### 3.3.2. Diagnosis/Treatment

The clinical signs and symptoms, along with radiographic evidence of periapical radiolucency and missed canals, led to a diagnosis of chronic apical abscess and failed endodontic treatment due to incomplete cleaning and obturation of complex canal systems.

**Figure 11:**
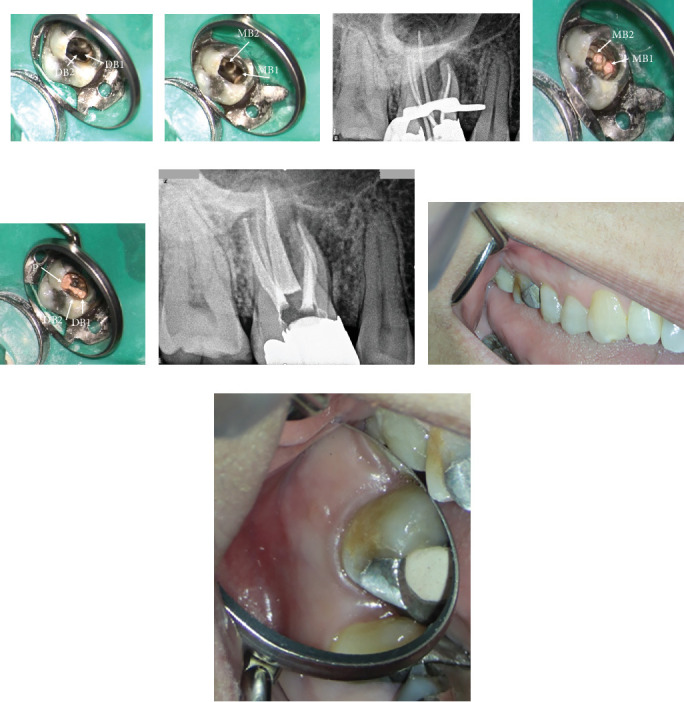
(a) Identification of missed DB2 canal. (b) Identification of missed MB2 canal. (c) Master cone fitness of all five canals. (d) The orifices of obturated MB1 and MB2 canals in the pulp floor. (e) The orifices of obturated DB1 and DB2 canals in the pulp floor. (f) Radiographic view of final obturation in five canals. (g, h) Clinical image showing resolution of the buccal swelling.

After obtaining informed consent, local anesthesia was administered using 2% lidocaine with epinephrine 1/80,000 (Darupakhsh, Tehran, Iran). The existing amalgam restoration was carefully removed to access the root canals using round bur number 2 (Jota AG, Rüthi, Switzerland). After rubber dam isolation, a DOM with magnification ranging from 4× to 15× (Magna, Labomed, Labo America Inc., United States) was used to enhance visualization. The previously obturated root canal filling material was meticulously removed from all canals using eucalyptol (Cerkamed, Poland) and Superfile III rotary instruments (Denco, Shenzhen, China). Upon inspection, two previously missed canals were identified: an additional distobuccal canal (DB2) and a second mesiobuccal canal (MB2) (Figure 11a,b). These findings correspond to the Ahmed et al. canal configuration: 3 MB 2-1-1, DB 2-2-2, P 1.

The canals were subsequently prepared using rotary instruments to achieve adequate cleaning and shaping, up to size 25 with 4% taper for all MB and DB canals and size 30 with 4% taper for the P canal. The canals were irrigated with 5.25% NaOCl to effectively remove debris and disinfect the canal system. After thorough cleaning and shaping, calcium hydroxide Ca(OH)2 paste (Merck, Darmstadt, Germany) was placed as an intracanal medicament in all canals to facilitate disinfection and promote healing of the periapical tissues. The tooth was temporarily restored with an IRM (Dentsply Sirona). At the subsequent appointment, the patient reported being asymptomatic with no signs of swelling or discomfort. Upon removal of the temporary restoration, the canals were irrigated again with NaOCl, saline, and EDTA 17% (Master-Dent, Dentonics Inc., United States) and agitated by a U-file ultrasonic tip size 15, 0.02 taper (NSK Varios, Nakanishi Inc., Tochigi, Japan) to ensure complete removal of the intracanal medicament and any remaining debris. The canals were dried using sterile paper points (META, Chungbuk, South Korea) and master cone fitness confirmed by radiography (Figure 11c). The final obturation was accomplished using lateral compaction techniques with GP (META, Chungbuk, South Korea) and AH-26 sealer (Dentsply, Tulsa Dental, Tulsa, Oklahoma, USA) to ensure complete filling of all identified canals. After sealing the access cavity with the IRM (Dentsply Sirona), postobturation radiographs confirmed successful obturation of five canals within the tooth, demonstrating adequate density and continuity of the filling (Figures 11d, 11e, and 11f).

### 3.4. Case 4 (Endodontic Management of a Maxillary Left First Molar With Complex Canal Anatomy)

#### 3.4.1. Case History/Examination

This case report presents the endodontic treatment of a 39-year-old female patient with no medical problem referred to a dental clinic due to pain and discomfort associated with her upper left first molar (Tooth #14). The patient's symptoms, combined with clinical and radiographic findings, indicated the presence of significant endodontic complications necessitating advanced treatment.

**Figure 12:**
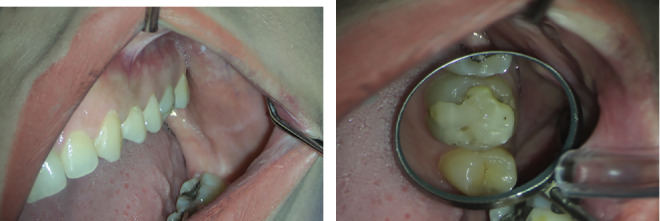
(a, b) Localized erythema and swelling of the soft tissue and alveolar mucosa surrounding Tooth #14.

The patient presented with localized pain in the upper left molar region, which had progressively worsened over the preceding weeks. Clinical examination revealed notable localized erythema and swelling of the soft tissue and alveolar mucosa surrounding Tooth #14 (Figure 12a,b).

Palpation and percussion tests indicated tenderness, suggesting underlying periapical pathology.

**Figure 13:**
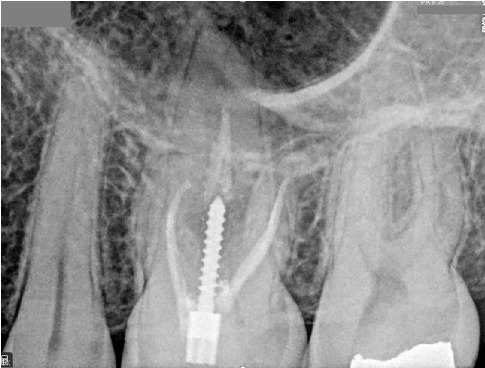
Previous endodontic treatment of Tooth #14 involved the placement of a prefabricated large post within the palatal canal, while all canals were inadequately prepared.

Radiographic evaluation revealed that Tooth #14 (maxillary left first molar) had undergone previous endodontic treatment, characterized by a large prefabricated post placed in the P canal (Figure 13).

**Figure 14:**
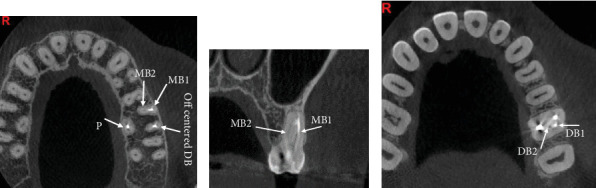
(a, b) Axial view of the CBCT revealing two distinct mesiobuccal canals (MB1 and MB2) along with an off-centered obturated distobuccal canal. (c) Coronal views of Tooth #15 highlighting the missed canals.

However, the treatment appeared inadequate, as evidenced by underprepared and poorly obturated canals. Notably, the MB canal exhibited severe curvature in the apical third, resulting in incomplete shaping and obturation. Similarly, the DB root demonstrated a severe curvature, leading to similar inadequacies in treatment. Additionally, radiographic images indicated periapical radiolucency surrounding both the P and DB canals, while widening of the periodontal ligament space was observed around the MB root. To further elucidate the complex canal anatomy and confirm the diagnosis, a CBCT scan was ordered. The axial view of the CBCT revealed that the MB canal had two distinct canals (MB1 and MB2) (Figure 14a), while the DB root also showed off-center obturated canals (Figure 14a,b), which indicates nonfilled spaces or another canal. Coronal views confirmed the presence of these missed canals, highlighting the complexity of the root canal system in this tooth (Figure 14c).

#### 3.4.2. Diagnosis/Treatment

**Figure 15:**
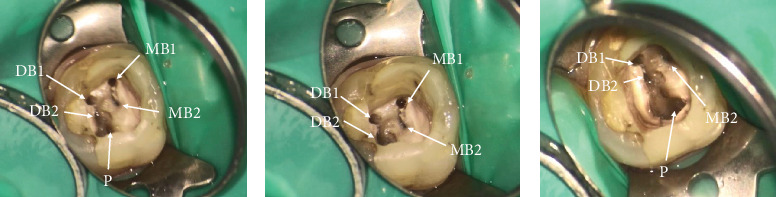
(a–c) Locating missed MB2 and DB2 canals in different angulations is demonstrated in intraoral photographs.

Upon obtaining informed consent, local anesthesia was administered to ensure patient comfort during the procedure with lidocaine 2% and epinephrine 1/80,000 (Darupakhsh, Iran). The existing composite restoration on Tooth #14 was carefully removed with a high-speed diamond round bur #2 (Jota AG, Rüthi, Switzerland) under 8× magnification using a DOM (Labomed Inc., United States, dental microscope). The previously placed post in the P canal was loosened using ultrasonic instrumentation (Varios, NSK, Japan) and subsequently removed. The tooth was then isolated with a rubber dam and clamp. After complete removal of GP from all identified canals using a rotary instrument and eucalyptol, additional canal scouting—specifically for MB2 and DB2—was initiated. Utilizing a 10× magnification setting on the DOM and an ultrasonic tip (NSK Varios, Nakanishi Inc., Tochigi, Japan) for thorough exploration of the pulp chamber, the previously missed canals were successfully located (Figures 15a, 15b, and 15c).

**Figure 16:**
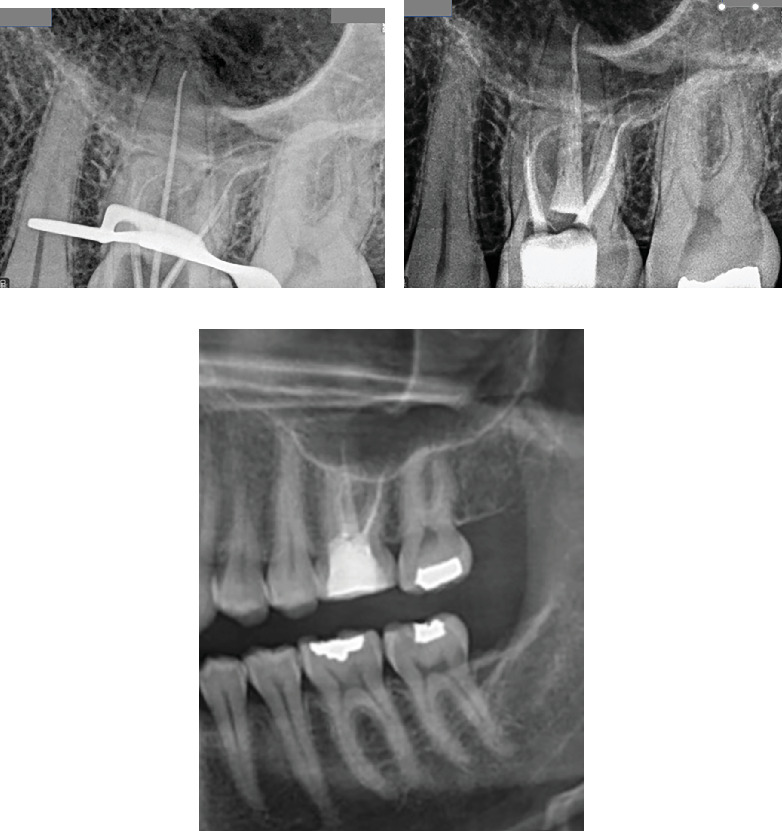
(a) Radiographic image illustrating the placement of the fitted master cone during endodontic treatment. (b) Postobturation radiograph confirming adequate filling of all root canals. (c) A segment of the panoramic radiographic view obtained at the 3-year follow-up reveals complete resolution of the previously identified periapical pathology, with no signs of recurrent lesions or abnormal periradicular changes.

The cleaning and shaping of all identified canals were initiated using rotary files NeoNiTi A1 #20 (Neolix, Châtres-la-Forêt, France) up to #25 following a crown-down technique. The canals were irrigated with 5.25% NaOCl to effectively remove debris and disinfect the canal system. To promote healing and disinfection, an intracanal medicament composed of calcium hydroxide Ca(OH)2 paste (Merck, Darmstadt, Germany) combined with CHX 2% (NikDarman, Iran) was placed within the canals. The access cavity was temporarily restored using IRM (Dentsply Sirona) to protect the tooth until the next appointment. At the subsequent appointment, the patient reported no signs of pain or discomfort upon palpation or percussion. Following the administration of local anesthesia, the temporary restoration was removed. After isolation with a rubber dam, the tooth was irrigated with copious amounts of NaOCl, normal saline, and EDTA 17% (Master-Dent, Dentonics Inc., United States), followed by ultrasonic agitation to enhance cleaning efficacy. After thorough drying of the canals using paper points (META, Chungbuk, South Korea), the lateral compaction obturation technique was performed due to the narrow root anatomy and the presence of multiple canals. NiTi spreaders, GP (META, Chungbuk, South Korea), and AH-26 sealer (Dentsply, Tulsa Dental, Tulsa, Oklahoma, United States) were used to achieve optimal apical sealing. The access cavity was then temporarily restored with IRM (Dentsply Sirona). Postobturation radiographs confirmed adequate filling of all identified canals. Based on radiographic assessment and in accordance with Ahmed et al. [18] canal coding system, the configuration was recorded as 14 MB2–1–1, DB 2–1–1, P1 (Figure 16a,b).

Panoramic radiographic evaluation at the 3-year follow-up reveals complete resolution of the previously identified periapical pathology, with no evidence of recurrent lesions or abnormalities in the periradicular structures (Figure 16c).

## 4. Review of the Literature

To assess the clinical significance of anatomical variations in maxillary molars, specifically the presence of additional canals such as the MB3 and the DB2, a systematic search was conducted across PubMed, Scopus, and Web of Science. The search employed keywords including “canal morphology,” “CBCT,” “DB2 canal,” “endodontics,” “maxillary molars,” and “MB3 canal.” Studies published from 1983 onwards, in both English and non-English languages, were considered. Selection criteria prioritized studies reporting on the prevalence and clinical outcomes related to missed MB3 and DB2 canals, while excluding articles focusing solely on traditional endodontic techniques or those lacking sufficient anatomical or clinical detail.

The literature reveals considerable variability in the reported prevalence of MB3 and DB2 canals, influenced by factors such as population demographics, sample size, and diagnostic methodologies. The summarized data from case reports and population studies are presented in Table 1, highlighting variations in canal morphology across different study populations and treatment contexts. This intricate anatomical variation was first described by Acosta Vigouroux and Trugeda Bosaans in 1978 (28, 50).

This table confirms that while the presence of MB2 is relatively common, the detection of MB3 and DB2 remains rare in maxillary molars. Reported prevalence rates for MB3 and DB2 canals generally remain below 5%, underscoring the need for meticulous diagnostic efforts [3].

Numerous case reports have documented anatomical variations in maxillary molars, with the number of canals ranging from two to five within a single tooth [6, 9, 16]. Demographic factors, including ethnicity and age, also influence canal morphology. Studies indicate that younger patients (typically aged 20–40) tend to have a greater incidence of accessory canals, attributed to less canal calcification, compared to older patients [31]. Geographic variability has been observed; for instance, some population-based evaluations from Iran [32] reported the absence of MB3 and DB2 canals, contrasting with findings from current case series within the same population demonstrating their presence. This highlights the need for patient-specific vigilance rather than reliance on generalized epidemiological data.

Despite technological advancements, MB3 and DB2 canals remain underreported in the literature. A review of scientific databases has revealed only a limited number of studies discussing these anatomical variations in detail [33]. This underreporting highlights the need for increased awareness and further research to ensure the comprehensive treatment of all potential canals.

## 5. Discussion

The case series presented herein demonstrates the clinical significance of MB3 and DB2 canals in maxillary molars and highlights the crucial role of advanced diagnostic tools in their detection and management. The use of CBCT and DOMs proved instrumental in identifying and treating previously missed canals, leading to superior clinical outcomes and underscoring their value in modern endodontic practice. For all patients in this study, comprehensive periodontal and restorative consultations were performed before initiating endodontic treatment to confirm the viability and restorability of the involved teeth. All teeth were clinically restorable and periodontally stable, ensuring a favorable prognosis.

In Case 1, four canals (MB1, MB2, DB, and P) were identified during the first appointment. While locating an MB2 canal in a maxillary second molar is already clinically uncommon, the presence of an MB3 canal was only detected during the second appointment by carefully examining the pulp chamber floor under a DOM and identifying pulp tissue remnants near the P canal orifice. The use of ultrasonic tips to remove calcifications in this area was critical for exposing the hidden canal orifice. This approach highlights the importance of thorough clinical exploration that combines magnification with ultrasonic instrumentation to uncover additional canals obscured by calcified dentin or complex anatomy, thereby enhancing the clinician's ability to locate and effectively treat all canals, improving treatment outcomes. Furthermore, carefully designed access cavity preparation is fundamental to allow adequate visualization and instrumentation of all canals [1]. An accurately executed access opening provides clear visualization of the pulp chamber floor, which is crucial for identifying all canal orifices, including often-hidden canals such as MB3 and DB2 [1, 20]. The shape and size of the access cavity should be adapted to the specific tooth morphology to ensure adequate canal access without compromising tooth structure. Techniques such as ultrasonic tips can refine the access cavity and remove obstructive dentin that may obscure canal orifices [1]. In our cases, the access cavity was modified according to the pulp chamber morphology, changing from the conventional triangular to a rhomboid shape—one of the key modifications for locating extra canals [34].

Accurate diagnosis of extra canals is crucial for successful endodontic treatment. Conventional radiographic techniques often fail to visualize canals such as MB3 and DB2 due to their small size and challenging anatomical positions [28]. This limitation underscores the necessity for advanced imaging and visualization methods. CBCT has significantly enhanced endodontic diagnostic capabilities by providing three-dimensional images that offer detailed visualization of the root canal system, substantially improving the detection of additional canals like MB3 and DB2 [1, 3–6, 9–12, 35]. The broad buccolingual dimension of the MB root may explain the presence of these accessory canals [11], a feature clearly demonstrated in axial CBCT slices from Case 2 (Figure 6a) and Case 4 (Figure 14a,b). These images confirm that root shape and cross-sectional anatomy allow precise identification of multiple canals within the MB and DB roots. The axial views help determine the number and location of canals along the corono-apical axis, while coronal views reveal the levels where canals split or merge [36], as shown in Case 2 (Figure 6b) and Case 4 (Figure 14c). Given this detailed anatomical information, CBCT is currently recommended, when indicated, to facilitate accurate detection of root canals in multirooted teeth.

Beyond advanced imaging, intraoperative visualization is crucial for successful endodontic treatment. Magnification provided by DOM greatly aids in locating and negotiating narrow or difficult-to-access canals, such as MB3 and DB2, which are often missed without enhanced vision [1, 10, 11, 13–16, 37]. The improved visualization allows for more precise instrumentation and obturation, reducing the risk of procedural errors [10]. In all four cases presented, DOM was utilized throughout treatment, emphasizing its essential role in managing complex canal anatomy. The combined use of CBCT and DOM proved highly effective: CBCT offers a comprehensive anatomical map of the root canal system, while DOM enables precise intraoperative navigation and manipulation. This synergistic approach greatly enhances the detection and treatment of all canals, contributing to improved clinical outcomes and minimizing persistent infections [10, 11, 16, 37].

A substantial body of research indicates a higher incidence of additional root canals in younger patients aged 20–40 years [31]. In our study, three of four cases involved patients aged 30–40, and the fourth, a 43-year-old, had initial treatment 10 years prior, aligning with the reported age range. Clinicians should remain vigilant with younger patients, as thorough exploration and advanced diagnostics enhance canal detection and treatment success.

The intricate anatomical variations in maxillary molars, especially within their MB roots, underscore the critical role of standardized classification systems in advancing both clinical practice and research communication. The classification framework proposed by Ahmed et al. effectively addresses this need by providing a systematic method for documenting root canal configurations [18]. This system is particularly advantageous when managing cases with multiple canals or complex morphologies [11, 18, 38]. Its structured approach significantly enhances diagnostic precision, as demonstrated in our findings: one case during initial treatment unveiled an additional canal, while three out of four retreatment cases identified canals previously undetected. These frequently overlooked canals, often sites of anatomical complexity, highlight the limitations of conventional diagnostic techniques. Applying a standardized classification system for root canal anatomy enables clinicians to better anticipate complex canal variations, leading to more thorough exploration and reduced incidence of missed canals. This approach facilitates tailored treatment strategies and improves communication and documentation, ultimately enhancing treatment planning and clinical outcomes by minimizing persistent infections due to untreated accessory canals [11, 18, 38].

Following treatment, all cases achieved complete resolution of pain. Notably, Cases #3 and #4 exhibited full healing of sinus tracts and elimination of purulent discharge. In Case #1, radiographic follow-up at 7 years and 4 months demonstrated normal periradicular tissues, confirming the successful identification and treatment of all root canals. At the 27-month follow-up, Case #2 exhibited complete resolution of the periapical radiolucency, while Case #4 showed similar favorable healing outcomes at the 3-year follow-up, both indicating sustained periapical tissue regeneration and long-term treatment success. These outcomes emphasize the importance of thorough identification and management of additional canals in maxillary molars, which significantly improve treatment success and long-term tooth retention. The interplay between anatomical oversight and systemic vulnerability is illustrated in Case #3, where hypothyroidism likely contributed to persistent periapical pathology by impairing immune response, while missed bacterial biofilms in untreated canals sustained inflammation. This clinical pattern was evident in three of the four initial treatments, all of which failed to detect additional canals, leading to symptomatic recurrences requiring retreatment.

In these cases, the clinician's expertise and meticulous use of advanced diagnostic tools such as CBCT and DOM were indispensable. Clinician proficiency in locating these elusive canals during retreatment was key to clinical success, as evidenced by complete pain resolution and successful follow-ups.

These findings highlight the paramount importance of clinician expertise combined with advanced imaging and magnification technologies—not only as reactive solutions during retreatment but as proactive measures during initial therapy. This approach is essential to overcome both biological challenges, such as compromised host defenses, and technical obstacles posed by complex root canal systems, ultimately improving clinical and radiographic prognosis in maxillary molars.

In addition to the systemic and anatomical complexities discussed, it is important to recognize that, in some retreatment cases, coronal seal failure can contribute to reinfection and treatment failure [39]. However, when missed canals are also present, treatment failure is almost certain [40]. The clinical significance of detecting and managing these additional canals is demonstrated by the resolution of clinical symptoms—such as swelling, pain, and purulent discharge—together with favorable long-term radiographic outcomes following their treatment. These findings emphasize the importance for clinicians to maintain a high level of suspicion for additional canals in maxillary molars, particularly in retreatment cases, and to perform a thorough exploration to ensure comprehensive debridement and long-term treatment success.

## 6. Limitations

Despite extensive efforts, follow-up access to one of the patients could not be obtained.

## 7. Conclusion

In conclusion, the successful identification and treatment of MB3 and DB2 canals in maxillary molars, as demonstrated in this case series, highlight the importance of advanced diagnostic tools and meticulous clinical techniques in endodontics. The integration of CBCT and DOM in clinical practice can significantly enhance the detection and management of complex root canal anatomies, leading to improved treatment outcomes and long-term successes. Knowledge and expertise are essential, and devices assist expert clinicians. As our understanding of root canal morphology continues to evolve, so too must our approach to endodontic diagnosis and treatment, emphasizing the need for continuous learning and adaptation of new technologies in clinical practice.

## Figures and Tables

**Table 1:** Previous reports of additional canals in maxillary molars.

**Year**	**Tooth number**	**Tx**	**Missed canal in retreatment**	**MB canal**	**DB canal**	**Root**	**Author**
1983	MFM	P		3	2	3	Martínez-Berná et al. [9]
1988	MFM	P		3	2	3	Bond et al. [20]
2009	MFM	P		3	2	3	De Almeida-Gomes et al. [21]
2010	MFM	P		3	2	3	Albuquerque et al. [22]
2010	MFM	P		3	2	3	Karthikeyan et al. [23]
2011	MFM	P		3	3	3	Kottor et al. [24]
2012	MFM	P		3	2	3	Sharath Chandra et al. [25]
2014	MFM	P		3	2	3	Gautam et al. [26]
2014	MFM	P		3	2	3	Badole et al. [13]
2017	MFM	P		3	3	3	Venumuddala et al. [14]
2019	MFM	P		3	1	3	Izaz Shaik et al. [15]
2021	MSM	P		3	2	3	Lin et al. [26]
2021	MFM	P		2	2	3	Lin et al. [26]
2021	MFM	Re	MB3, DB2	3	2	3	Kumar et al. [27]
2022	MFM	P		3	2	3	Rashmi et al. [28]
2024	MFM	P		3	2	3	Kadulkar et al. [10]
2024	MFM	P		3	2	3	Arnold and Ahmed [11]
2024	MFM	P		3	2	3	Kadoo et al. [12]
2024	MFM	P		2	2	3	Alghamdi et al. [29]
2024	MSM	P		3	1	3	Liu et al. [16]
2025	MSM	P		2	2	3	Zerafat et al. [30]
Case 1 Case 2 Case 3 Case 4	MSM MFM MFM MFM	P Re Re Re	MB1 MB2, DB2 MB2, DB2	3 3 2 2	1 1 2 2	3 3 3 3	Aminsobhani and Majidi in the present cases

Abbreviations: MFM, maxillary first molar; MSM, maxillary second molar; P, primary treatment; Re, retreatment.

## Data Availability

The data supporting the findings of the present study are available from the corresponding author upon request.
